# Reductions in smoking due to ratification of the Framework Convention for Tobacco Control in 171 countries

**DOI:** 10.1038/s41591-024-02806-0

**Published:** 2024-02-06

**Authors:** Guillermo Paraje, Mauricio Flores Muñoz, Daphne C. Wu, Prabhat Jha

**Affiliations:** 1 Business School, Universidad Adolfo Ibáñez, Santiago, Chile; 2 Millennium Nucleus for the Evaluation and Analysis of Drug Policies, Santiago, Chile; 3 Universidad Adolfo Ibáñez, Santiago, Chile; 4 Centre for Global Health Research, Unity Health Toronto and Dalla Lana School of Public Health, University of Toronto, Toronto, Ontario Canada

**Keywords:** Risk factors, Epidemiology

## Abstract

Smoking globally kills over half of long-term smokers and causes about 7 million annual deaths. The World Health Organization Framework Convention for Tobacco Control (FCTC) is the main global policy strategy to combat smoking, but its effectiveness is uncertain. Our interrupted time series analyses compared before- and after-FCTC trends in the numbers and prevalence of smokers below the age of 25 years (when smoking initiation occurs and during which response to interventions is greatest) and on cessation at 45–59 years (when quitting probably occurs) in 170 countries, excluding China. Contrasting the 10 years after FCTC ratification with the income-specific before-FCTC trends, we observed cumulative decreases of 15.5% (95% confidence interval = −33.2 to −0.7) for the numbers of current smokers and decreases of −7.5% (95% CI = −10.6 to −4.5) for the prevalence of smoking below age 25 years. The quit ratio (comparing the numbers of former and ever smokers) at 45–59 years increased by 1.8% (1.2 to 2.3) 10 years after FCTC ratification. Countries raising taxes by at least 10 percentage points concurrent with ratification observed steeper decreases in all three outcomes than countries that did not. Over a decade across 170 countries, the FCTC was associated with 24 million fewer young smokers and 2 million more quitters.

## Abstract (web summary)

An interrupted time series analysis found that the World Health Organization Framework Convention for Tobacco Control reduced smoking prevalence across 170 countries (excluding China) since the country-specific year of ratification.

## Main

Prolonged tobacco smoking kills more than half of long-term smokers, leads to an average of a decade of life lost for individual smokers^1^ and contributes to over 7 million tobacco deaths annually globally^2^. In high-income countries (HICs), one death can be expected for approximately every million cigarettes consumed^3^. Despite declines in adult smoking prevalence recorded in most countries over the last decade, the absolute number of tobacco deaths continues to grow in many lower- and middle-income countries (LMICs), where most the world’s 1.1 billion smokers in 2019 reside. Increases in absolute tobacco deaths are fueled by population growth, but also by the earlier onset of smoking uptake among smokers and the emergence of the full effects of prolonged smoking from young ages among the increasing number of adults reaching middle and older age^4,5^.

The main global strategy to reduce smoking has been the World Health Organization (WHO)-led Framework Convention for Tobacco Control (FCTC), a global treaty with a comprehensive set of measures to decrease smoking, including demand reduction, countering smuggling, protection from exposure to tobacco smoke, regulation of tobacco product contents, packaging and labeling, as well as regulating tobacco advertising, promotion and sponsorship. The FCTC drew on evidence developed in partnership with the World Bank, which identified that the single most effective intervention was to raise excise taxation (that is, taxes levied on specific products or services) on tobacco products^6^. The FCTC was adopted in 2003 and became legally binding as an international treaty in 2005. In 2008, WHO adopted an MPOWER strategy to help implement the key FCTC provisions^7^.

The extent to which the FCTC and the follow-up MPOWER strategies have reduced smoking in LMICs is debatable^8,9^, with most evaluations relying on several limiting assumptions^8,10^ or only tracking MPOWER coverage^11^. Variability in surveys used to determine smoking prevalence at various ages, changing income levels and concurrent changes in several tobacco control policies mean that typical regression analyses across countries are of limited value to evaluate if ratification of the FCTC reduced smoking, and cannot easily quantify the role of taxation^12^.

A robust method to examine the impact of the FCTC is interrupted time series analysis (ITSA), which uses the background trends in consumption for each country compared to the after-FCTC period to assess the impact of the FCTC. ITSA is the preferred analytic method where randomization is not possible^13,14^. We applied ITSA to compare before and after ratification trends (accounting for the nonlinearity of smoking prevalence) for the number and prevalence of smokers at ages 10–24 years (because this age group will be more responsive to price and non-price interventions)^15^ as well as cessation among older adults aged 45–59 years (when most quitting efforts by smokers have been completed)^16,17^. We incorporated variation in ratification years (range 2004–2010; median 2005) and considered time to ratification and variability in the concurrent use of excise taxes to raise prices in the analyses. Table 1 summarizes our findings and policy implications.

**Table 1:** Policy summary

Section	Description
Background	Prolonged tobacco smoking kills more than half of long-term smokers, leads to an average of a decade of life lost for individual smokers and contributes to over 7 million tobacco deaths annually globally.
	The WHO FCTC is the main global policy strategy to combat smoking, although previous studies, some of them relying on limited assumptions, cast doubts on its effectiveness.
Main findings	At the global level (excluding China) after FCTC ratification in 170 countries, the prevalence of current smokers below age 25 decreased at an average annual rate of −0.8% (95% CI = −1.0 to −0.5), compared to the pre-FCTC period. The global (excluding China) post-FCTC trend for the quit ratio (ratio between former and ever smokers) at ages 45–59 years increased at an annual average of 0.1% (0.1–0.2). Countries that increased tobacco taxes by 10 percentage points or more after ratification reduced prevalence at an annual rate of −2.1% (−2.8 to −1.4) compared with those with little (below 10 percentage points) or no tax change, which had a reduction of −0.7% (−1.3 to −0.2). The global post-FCTC trend for the quit ratio at ages 45–59 years increased at an annual average in high-tax change countries by 0.5% (0.4 to 0.6) in contrast to a decrease of 0.1% (−0.2 to −0.1) for low-tax change countries.
	Globally (excluding China), after 10 years of FCTC ratification, there were 24.0 (0.4–47.6) million fewer smokers below age 25 or 15.5% of projected (before-FCTC trends) smokers. Additionally, there were 2.0 (1.4–2.6) million more quitters at 45–59 years because of increased cessation.
Limitations	Limitations of this study include the grouping of males and females in the analyses and the lack of analyses for individual countries, which precludes considering country-level covariates (for example, per capita income, changes in cigarette affordability).
Policy implications	We demonstrate that the FCTC, over only one decade after ratification, led to a statistically significant acceleration of declines in the overall number of smokers and in smoking prevalence below age 25 and an increase in relative cessation. Countries with moderate or large tobacco tax increases after ratification were more successful in curbing consumption. Countries must ratify FCTC, increase tobacco taxes and apply other non-price policies to reduce the enormous disease burden caused by tobacco.

## Results

### The effect of FCTC ratification on the number of current smokers and the quit ratio

We studied 171 countries, representing 89% of the global population in 2021. Supplementary Table 1 provides information on the average number and prevalence of current smokers during the 10 years before and after ratification, the year of FCTC ratification for each country, the total tobacco tax share for the most sold cigarette brand in 2008 and its change in percentage points in the 2008–2012 period, as well as an assessment of the quality of survey coverage^4,18^. China constitutes about one-third of global tobacco smokers, with most tobacco sold through state-owned companies^19^. Hence, we considered China separately in the analyses, excluding it from the global analyses (for example, World in the tables) and the ones conducted using World Bank income groups^20^.

Among 170 countries in four income regions and excluding China, in the year of FCTC ratification there were 153 million smokers below age 25 years, and 79 million former and 231 million ever smokers at ages 45–59 years (yielding a quit ratio of former to ever smokers of 0.34; Table 2). The prevalence of current smoking below age 25 years in the decade after FCTC ratification showed significant decreases compared to before ratification (95% confidence interval (CI) = 13.1 to 13.5, before ratification versus after ratification 95% CI =10.8 to 11.6). In most countries (109 of 171), the peak year of smoking prevalence was before FCTC ratification (data not shown)^21^, which we considered in the ITSA to establish before and after ratification trends.

**Table 2:** Prevalence before, after and at FCTC ratification in a population below age 25 years, according to country income and tax change groupings

Region (number of countries)	Number of smokers in the ratification year in millions		Evolution of prevalence of current smoking population below the age 25 years (95% CI)		
	Current smokers below the age of 25 years	Former or ever smokers aged 45–59 (and quit ratio^a^)	Prevalence 10 years before ratification	Prevalence at the year of ratification	Prevalence 10 years after ratification
World (170)^b^	153	79/231 (0.34)	13.3% (13.1 to 13.5)	12.5% (8.2 to 16.8)	11.2% (10.8 to 11.6)
China	35	10/86 (0.12)	10.8% (10.1 to 11.5)	10.7% (9.5 to 12.2)	13.1% (12.6 to 13.7)
**According to income group**^**c**^					
High (52)	34	33/79 (0.42)	24.9% (24.5 to 25.4)	23.1% (14.1 to 32.0)	20.6% (19.9 to 21.4)
Upper middle (50)^a^	46	25/65 (0.38)	19.2% (19.0 to 19.4)	18.0% (0.9 to 27.0)	15.9% (15.3 to 16.5)
Lower middle (47)	64	20/81 (0.24)	8.9% (8.9 to 9.0)	8.9% (2.1 to 15.8)	8.4% (8.2 to 8.7)
Low (21)	9	2/7 (0.31)	8.7% (8.7 to 8.8)	8.6% (5.8 to 11.4)	8.2% (7.9 to 8.4)
**According to tax change**^**d**^					
High-tax change (23)	24	10/36 (0.28)	18.7% (18.5 to 18.9)	17.6% (4.7 to 30.4)	14.5% (13.6 to 15.4)
Low-tax change (137)	129	69/193 (0.36)	12.6% (12.4 to 12.8)	11.9% (6.7 to 17.1)	10.8% (10.4 to 11.2)

^a^Quit ratio is the former to ever smoker ratio as a measure of cessation.

^b^Excluding China.

^c^All subsequent analyses exclude China.

^d^One hundred and sixty countries for which data were available (excluding China).

95% CIs were obtained using bootstrapping (100 replications).

We used the ITSA to examine the before-FCTC and after-FCTC trends in the logarithm of the number of smokers, the prevalence at below age 25 years and the quit ratio at ages 45–59 years. We examined trends as the logs of respective values, given the marked skew in the data, to examine relative changes in the before- and after-FCTC periods. Hence, all the results are presented as log values unless otherwise stated.

The main analyses showed that the after-FCTC trends fell faster in relation to the before-FCTC trends (Table 3). At the global level, after FCTC ratification in 170 countries, the prevalence of current smokers below age 25 years decreased at an average annual rate of −0.8% (95% CI = −1.0 to −0.5) compared to the before-FCTC period (Table 3). The sharpest declines in after-FCTC trends were in low-income countries (LICs) where the number and prevalence of current smokers decreased at an annual average rate of −6.0% (95% CI = −8.1 to −4.0) and −4.0% (95% CI = −5.6 to −2.4), respectively. By contrast, the slope of the after-FCTC trend in prevalence increased at an average annual rate of 1.3% (95% CI = 0.6 to 1.9 in HICs in relation to its before-FCTC trend, which did not prevent an actual decrease in prevalence, as seen in Table 2). The global after-FCTC trend for the quit ratio at ages 45–59 years increased at an annual average of 0.1% (95% CI = 0.1 to 0.2). The after-FCTC trend for the quit ratio increased more in high-income and LMICs, but paradoxically decreased by −0.2% (95% CI = −0.2 to −0.1) in LICs. Full results of the models showing changes in before- and after-FCTC trends and changes in levels with the FCTC are shown in Supplementary Tables 3–5.

**Table 3:** Average annual change in trends after WHO FCTC ratification compared to pre-FCTC trends in the logarithm of the number of smokers and prevalence in the population below age 25 years, and the logarithm of the quit ratio for the population aged between 45 and 59 years, according to country income and tax change groupings

Region (number of countries)	Cumulative effect (10 years) after ratification (best fitted model^a^), % and (95% CI)		
	Number of smokers below age 25 years	Prevalence below age 25 years	Quit ratio ages 45–59 years
World (170)^a^	−1.6 (−3.1 to −0.1)^f^	−0.8 (−1.0 to −0.5)^d^	0.1 (0.1 to 0.2)^c^
**According to income group**			
High (52)	−0.1 (−0.3 to 0.1)^e^	1.3 (0.6 to 1.9)^f^	0.2 (0.1 to 0.2)^c^
Upper middle (50)^b^	−3.3 (−7.2 to 0.5)^f^	−1.0 (−1.5 to −0.6)^e^	−0.2 (−1.0 to 0.6)^f^
Lower middle (47)	−0.8 (−1.0 to −0.7)^e^	−0.7 (−0.8 to −0.5)^e^	0.1 (0 to 0.3)^c^
Low (21)	−6.0 (−8.1 to −4.0)^f^	−4.0 (−5.6 to −2.4)^f^	−0.2 (−0.2 to −0.1)^c^
**According to tax change**			
High-tax change (23)	−2.7 (−3.6 to −1.8)^e^	−2.1 (−2.8 to −1.4)^e^	0.5 (0.4 to 0.6)^e^
Low-tax change (137)	−1.2 (−1.4 to −1.0)^d^	−0.7 (−1.3 to −0.2)^f^	−0.1 (−0.2 to −0.1)^c^

^a^A list of the five possible ITSA models is provided in the Methods.

^b^Excluding China.

^c^Model 1 corresponds to before and after intervention linear trends. Model 2 corresponds to a before intervention polynomic quadratic trend and an after intervention linear trend.

^d^Model 3 corresponds to before and after intervention polynomic quadratic trends.

^e^Model 4 corresponds to a before intervention polynomic cubic trend and an after intervention linear trend.

^f^Model 5 corresponds to a before intervention polynomic cubic trend and an after intervention polynomic quadratic trend.

### Combined effects of FCTC ratification and tax increases

Only 23 of the 161 countries for which we had taxation data were classified as high-tax change, meaning that the tax burden on the most commonly sold cigarette brand rose by at least 10 percentage points along with FCTC ratification. These high-tax change countries had a higher prevalence of current smokers below age 25 years in the before-FCTC trends compared to countries with low-tax change (18.7% versus 12.6%), which continued in the after-FCTC period (14.5% versus 10.8%) (Table 2). At these ages, the after-FCTC trends in countries with high-tax change decreased the number and prevalence of smokers at an annual average rate of −2.7% (95% CI = −3.6 to −1.8) and −2.1% (95% CI = −2.8 to −1.4), respectively. These decreases were approximately twice as large as those observed in low-tax change countries for the number (−1.2, 95% CI = −1.4 to −1.0) and prevalence (−0.7, 95% CI = −1.3 to −0.2) of smokers, respectively. The global after-FCTC trend for the quit ratio at ages 45–59 years increased at an annual average in high-tax change countries of 0.5% (95% CI = 0.4 to 0.6) in contrast to a decrease of 0.1% (95% CI = −0.2 to −0.1) for low-tax change countries.

### Cumulative effects over 10 years

**Fig. 1:**
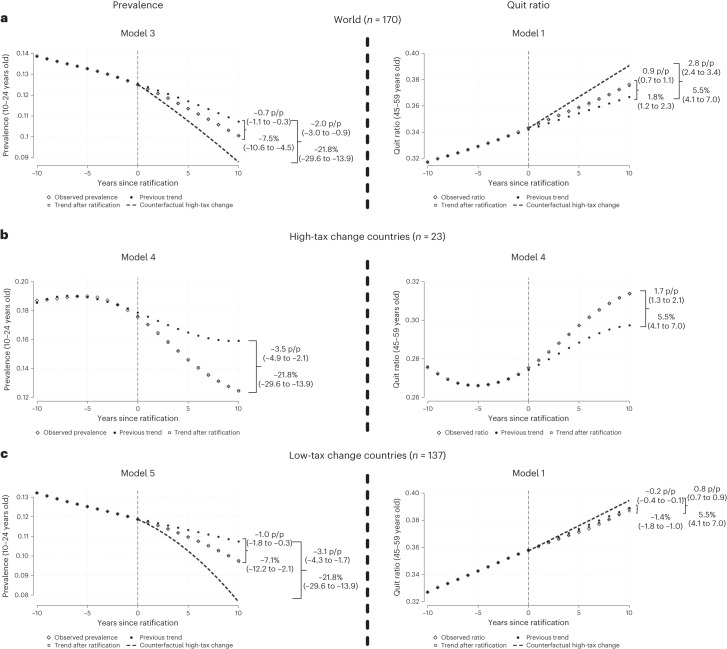
Evolution of the before- and after-FCTC trends for the prevalence of current smoking below age 25 and the quit ratio for the population aged between 45 and 59 years. **a**–**c**, The expected decline worldwide (**a**) and in low-tax change countries (**c**) represents the proportionate decline seen in high-tax change countries (**b**) (where the tax burden rose at least 10 percentage points between 2008 and 2012). Shown are the percentages, relative prevalence and quit ratio decrease or increase 10 years after ratification, along with the 95% CIs. Note: China is excluded.

The global cumulative effect 10 years after ratification meant an after-FCTC decrease of −15.5% (95% CI = −33.2 to −0.7) for the numbers and a decrease of −7.5% (95% CI = −10.6 to −4.5) for the prevalence of current smokers below age 25 years (Fig. 1), in relation to the cumulative effect that would have occurred at the before-FCTC trends (Table 4). The largest decreases occurred in LICs, followed by upper-middle-income countries; the smallest decreases occurred in HICs. The global cumulative increase in the quit ratio at ages 45–59 years was 1.8% (95% CI = 1.2 to 2.3), with the largest increase in HICs, followed by LMICs, with LICs showing a slight increase in quit ratio. Similarly, the decreases in after-FCTC trends in the number and prevalence of smokers and the increases in quit ratio were greater in countries with higher tax increases than in countries with low or no tax increases.

**Table 4:** Cumulative effect 10 years after WHO FCTC ratification in the logarithm of the number of smokers and prevalence for a population below age 25 years and the logarithm of the quit ratio for a population between 45 and 59 years old, according to country income and tax change groupings

Region (number of countries)	Cumulative effect (10 years) after ratification (best fitted model^a^), % and (95% CI)		
	Logarithm of the number of smokers below age 25 years	Logarithm of prevalence below age 25 years	Logarithm of the quit ratio ages 45–59
World (170)^a^	−15.5 (−33.2 to −0.7)^f^	−7.5 (−10.6 to −4.5)^3^	1.8 (1.2 to 2.3)^c^
**According to income group**			
High (52)	−1.9 (−4.2 to 0.4)^e^	11.5 (5.3 to 17.7)^f^	1.5 (1.1 to 1.9)^c^
Upper middle (50)^a^	−32.3 (−69.1 to 4.5)^f^	−11.0 (−15.8 to −6.1)^e^	−1.1 (−8.7 to 6.6)^f^
Lower middle (47)	−8.4 (−9.9 to −6.8)^e^	−6.8 (−8.0 to −5.6)^e^	0.7 (−0.2 to 1.6)^c^
Low (21)	−54.9 (−74.5 to −35.4)^f^	−36.8 (−52.2 to −21.5)^f^	−1.7 (−2.3 to −1.1)^c^
**According to tax change**			
High-tax change (23)	−28.4 (−37.8 to −18.9)^e^	−21.8 (−29.6 to −13.9)^e^	5.5 (4.1 to 7.0)^e^
Low-tax change (137)	−11.5 (−13.6 to −9.4)^d^	−7.1 (−12.2 to −2.1)^f^	−1.4 (−1.8 to −1.0)^c^

^a^A list of the five possible ITSA models is provided in the Methods.

^b^Excluding China.

^c^Model 1 corresponds to before and after intervention linear trends. Model 2 corresponds to a before intervention polynomic quadratic trend and an after intervention linear trend.

^d^Model 3 corresponds to before and after intervention polynomic quadratic trends.

^e^Model 4 corresponds to a before intervention polynomic cubic trend and an after intervention linear trend.

^f^Model 5 corresponds to a before intervention polynomic cubic trend and an after intervention polynomic quadratic trend.

**Fig. 2:**
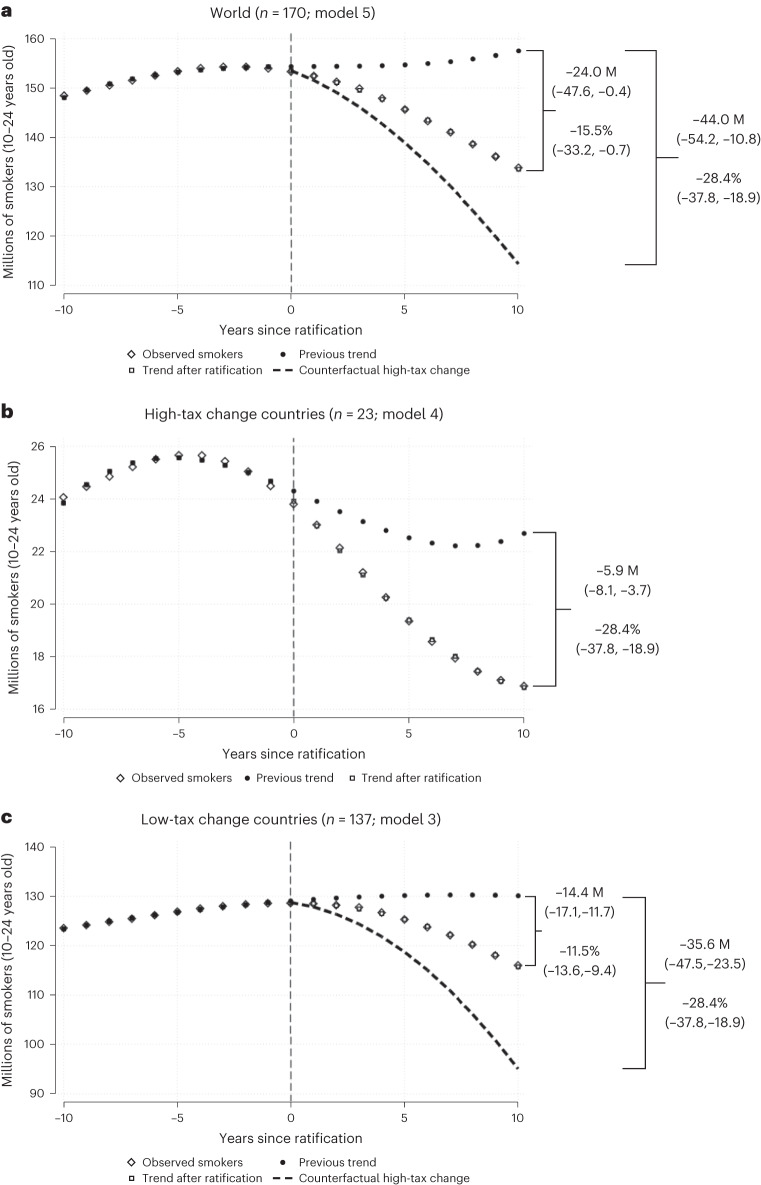
Evolution of before- and after-FCTC trends for the number of current smokers in a population aged below 25 years. **a**–**c**, The expected decline worldwide (**a**) and in low-tax change countries (**c**) represents the proportionate decline seen in high-tax change countries (**b**) (where the tax burden rose at least 10 percentage points between 2008 and 2012). Decrease in the number of smokers and relative reduction of the number of smokers 10 years after ratification with 95% CIs. Note: China is excluded.

We projected the before-FCTC trends for current smokers for the 10 years after FCTC ratification and compared these projections to the actual number of current smokers during the same period to estimate the reduction in the number of smokers after FCTC ratification. Globally (excluding China), over these 10 years there were 24.0 (0.4–47.6) million fewer smokers below age 25 years or 15.5% of projected smokers (Fig. 2). In addition, over the decade after FCTC ratification, there were 2.0 (1.4–2.6) million more quitters at 45–59 years because of increased cessation (Supplementary Table 9). Had all countries increased the excise tax rate by at least 10 percentage points, matching high-tax change countries, there would have been 44 (10.8–54.2) million fewer smokers below age 25 years and 5.2 (4.0–6.9) million more quitters at ages 45–59 years.

## Discussion

We demonstrated that the FCTC, over only one decade after ratification, led to a statistically significant acceleration of decline in the overall number of smokers and in smoking prevalence below age 25 years, leading to about 24 million fewer smokers in this age group, consisting of 15.5% of projected smokers, and about 2 million more quitters at 45–59 years because of cessation. The relative decrease in the numbers of smokers and prevalence and increase in cessation were at least twice as large in the 23 countries that combined FCTC ratification with substantial tax increases during 2008–2012. The results also show that while the decrease in prevalence was stronger in LMICs after FCTC ratification, the decrease in cessation was more pronounced in HICs, which is consistent with previous studies^22^.

Among young smokers below age 25, cessation is uncommon in most LMICs^3,4,19^. Under conservative assumptions, it is estimated that at least half (and perhaps closer to two-thirds) of the 24 million averted younger smokers would die from smoking-related causes^23^. Thus, it is estimated that the FCTC could avoid at least 12 million eventual deaths from just one decade of implementation from the combined effects of reduced initiation and cessation.

Our analyses showing stronger effects of FCTC ratification in high-tax change countries do not directly show the effectiveness of tobacco taxes in curbing consumption but are consistent with the evidence that large increases in excise taxation of cigarettes are the single most effective intervention to reduce smoking initiation and raise cessation^24^. Although we did not demonstrate that taxation is the only policy responsible for the decrease in prevalence and the relative increase in cessation, we found that countries that made a stronger effort to curb consumption after FCTC ratification (proxied by a higher increase in tobacco taxes) were more successful in curbing consumption. We also showed that if low-tax change countries had effects after FCTC ratification comparable to high-tax change countries, there would have been 44 million fewer smokers below age 25 years and far more adult cessation globally.

Large excise tax increases, in the range of 100%, have been practicable in many settings^25^; indeed, a large increase may signal the importance of tobacco control to smokers^26,27^. The combination of FCTC ratification, with its publicity of smoking hazards and the adoption of non-price interventions, probably complemented the actual effect of raised prices. By contrast, China stood out with paradoxical increases in smoking after FCTC ratification. However, China’s market is unique because state-owned tobacco companies mostly supply it, with a vested interest in not dropping consumption^28,29^. Moreover, China has diverse cigarette brands priced at various levels that allow substitution by consumers and manufacturers^30^.

For most countries, the decrease in the number (and prevalence) of current smokers began before the ratification of the FCTC. At the time of ratification, the number of current smokers (and prevalence) was already stable or decreasing. However, our analyses show that ratification accelerated the decline in current smokers and prevalence below age 25 years and accelerated cessation among older smokers. An exception to this is HICs, where the decline in prevalence decelerated with ratification. In countries where tobacco taxes were increased substantially, such an acceleration in the reduction of smokers was higher, resulting in a more important number of smokers (and deaths) averted.

The results shown in this article must be examined within the broader context of tobacco control efforts. Non-price interventions, notably bans on smoking in public places, have expanded considerably, as noted in the 2023 WHO report on tobacco control^11^. This measure is highly cost-effective in reducing the disease burden from tobacco^31^ and increases the costs of smoking (by raising the time and convenience costs of smoking in public places). Ample evidence suggests that higher excise taxes are the single most effective intervention to deter young people from starting smoking while raising adult cessation^24,25^.

The ITSA is regarded as a robust evaluation tool^13,14,32^, but it has limitations. The robustness of the ITSA depends in part on establishing the actual underlying before-FCTC trends, which we have quantified by selecting bespoke models with linear and nonlinear trends using a well-established method. Our ITSA results contradict an earlier ITSA of the impact of the FCTC, which relied on using 2003 as the intervention year, even though the actual year of ratification varied^8^. Moreover, the earlier ITSA relied on per capita cigarette use back to 1970, which should not greatly influence the consumption trends decades later. In our ITSA, all countries except for China showed decreases in prevalence, which is consistent with secular trends due to greater awareness of tobacco hazards globally^18^. A natural strength of the ITSA is the ability to distinguish intervention effects, such as FCTC ratification, from these secular trends. We conducted additional sensitivity analyses looking at longer before ratification periods and variation in the ratification year, but these yielded similar results to our main analyses. Our grouping of male and female smokers together precluded the ability to examine cessation differences according to sex in key regions, such as East and South Asia, where there are fewer female smokers^2,33^. We did not examine individual countries (aside from China) but grouped them, which precludes considering country-level covariates (for example, per capita income, changes in cigarette affordability). Brazil’s rapid decline in smoking, which arose in part from much higher excise taxes^34,35^ may dominate the upper-middle-income category because it is a populous country of 215 million people.

The data used for the analyses are a widely published dataset, where such data are constructed after reviewing and analyzing many national surveys and modeling years or countries with no surveys. Countries with more surveys have more accurate information about the evolution of smoking prevalence and cessation. We classified countries with more than ten surveys in the 1990–2020 period as high-quality. Results using just countries with high-quality surveys were similar to those presented in this study (Supplementary Table 2).

The FCTC, while effective, is at risk of stalling^9^. The number of countries raising taxes has slowed and cigarette affordability has risen^36^. For example, India has had no notable tax increases for the last few years (at least since 2014)^37^. In India, the tobacco tax share of the most sold brand decreased between 2008 and 2012 after FCTC ratification; although it later increased, it is still well below the WHO recommendation of a tax share of at least 75% of the retail price^2,38^. WHO’s 2023 tobacco control report also documents the slowest progress in large increases in taxation^2^.

Our findings should help accelerate tobacco control by showing that the FCTC is effective, and within it, taxation is the key to future progress. Large tax increases might well avoid about 200 million deaths over the next few decades and are the only practicable way to achieve the targets for reduced chronic diseases within the United Nations Sustainable Development Goals^23,39,40^.

## Methods

### National smoking surveys and definitions

We derived data on the number of smokers and smoking prevalence among individuals aged 10–24 years from datasets produced by the Global Burden of Disease, Injuries, and Risk Factors (GBD) study, which relied on 3,625 nationally representative surveys on tobacco use for 204 countries and territories from 1990 to 2020 (refs. ^4,18^). The dataset provides annual estimates of smoking prevalence in 5-year age groups and the number of current and former smokers, with detailed methods already published^4,18^. The dataset is the result of a complex process of assessing and reviewing existing surveys, extracting relevant data from them; when there was no surveys for specific years or countries, modeling using spatiotemporal Gaussian process regressions was used^4^.Naturally, this process may imply smoothed data that can affect estimations in quasi-experimental settings^41^. For countries with more surveys, it would be expected that this limitation would be less stringent. We conducted separate analyses for countries with more than 10 years of surveys, which we defined as higher-quality data (Supplementary Table 2).

The surveys’ current or former smoking definitions varied but generally conformed to WHO’s standard definitions. Current smoking included daily use of any smoked tobacco (mostly manufactured cigarettes, but also hand-rolled cigarettes, cigars, cigarillos, pipes and other products)^18^. Former smokers were those who had smoked more than 100 cigarettes in their lifetime but were not current smokers. We calculated the number of current and former smokers by multiplying the annual age-specific and sex-specific prevalence estimates by the respective population in the GBD study.

We classified the 161 countries with available tax data (including China) according to changes in cigarette tax incidence between 2008 and 2012, using data from WHO annual reports (compiled from standardized surveys of each country)^5^. We classified countries as high-tax change countries if the tax burden on the retail price of the most commonly sold cigarette brand increased by 10 or more percentage points between 2008 and 2012 (almost all for substantial increases in excise taxation)^42^ and countries as low-tax change countries if the tax burden rose by less than 10%, remained unchanged or decreased. We adopted this classification to separate countries that ratified the FCTC and mostly concurrently substantially increased the tax burden on cigarettes from those that did not. We used 2008–2012 because 95% of countries ratified the FCTC before 2012 and because 2008 is the first year captured in WHO annual reports on tobacco taxation.

### Study design

We applied the ITSA, which uses a before–after design, to quantify the effect of FCTC ratification on the trends and levels of the logarithm (to account for the marked skew in the data and to examine the relative changes) of the number and prevalence of current smokers below age 25 years, on the assumption that the logarithm of these numbers should fall. We focused on this age group, given that most smokers who will be lifelong smokers initiate by age 25, and because more limited experimentation with quitting also occurs in these age groups. Moreover, younger adults are more price-sensitive to any increases in taxation recommended by the FCTC^43^. We also examined the logarithm of the quit ratio (former over ever smokers aged 45–59 years), which should also fall if cessation increases^22,44^. We chose this age because cessation efforts by smokers have largely been completed by age 60 (refs. ^16,17^).

Details on the ITSA methodology have been published elsewhere^13,45,46^; Supplementary Fig. 1 provides a schema for its operation, including the links to several model specifications used. We grouped countries according to the 2021 World Bank income grouping (low-, upper-middle, lower-middle and high-income)^47^, according to each country’s FCTC ratification year^18^.

The main analysis compares the change in annual trends after FCTC ratification, examining if the 10-year rates of decline before ratification in current smokers or prevalence further accelerated after ratification. For cessation, the main comparison examines whether the current to former smokers ratio rose after FCTC ratification. We also examined changes in level for both outcomes. However, an accelerated decline in current smoking (or increases in cessation) will most strongly determine the future course of tobacco deaths^48^ and it is thus the focus of our analyses.

### Statistical analysis

We estimated several ITSA models to consider before and after ratification trends (non)linearity. Concretely, we estimated the following models^46^:

1 $${Y}_{t}={{{\beta }}}_{0}+{{{\beta }}}_{1}{T}_{t}+{{{\beta }}}_{4}{X}_{t}+{{{\beta }}}_{5}{X}_{t}{I}_{t}+{{{\epsilon }}}_{t}$$

2 $${Y}_{t}={{{\beta }}}_{0}+{{{\beta }}}_{1}{T}_{t}+{{{\beta }}}_{2}{T}_{t}^{2}+{{{\beta }}}_{4}{X}_{t}+{{{\beta }}}_{5}{X}_{t}{I}_{t}+{{{\epsilon }}}_{t}$$

3 $${Y}_{t}={{{\beta }}}_{0}+{{{\beta }}}_{1}{T}_{t}+{{{\beta }}}_{2}{T}_{t}^{2}+{{{\beta }}}_{4}{X}_{t}+{{{\beta }}}_{5}{X}_{t}{I}_{t}+{{{\beta }}}_{6}{X}_{t}{I}_{t}^{2}+{{{\epsilon }}}_{t}$$

4 $${Y}_{t}={{{\beta }}}_{0}+{{{\beta }}}_{1}{T}_{t}+{{{\beta }}}_{2}{T}_{t}^{2}+{{{\beta }}}_{3}{T}_{t}^{3}+{{{\beta }}}_{4}{X}_{t}+{{{\beta }}}_{5}{X}_{t}{I}_{t}+{{{\epsilon }}}_{t}$$

5 $${Y}_{t}={{{\beta }}}_{0}+{{{\beta }}}_{1}{T}_{t}+{{{\beta }}}_{2}{T}_{t}^{2}+{{{\beta }}}_{3}{T}_{t}^{3}+{{{\beta }}}_{4}{X}_{t}+{{{\beta }}}_{5}{X}_{t}{I}_{t}+{{{\beta }}}_{6}{X}_{t}{I}_{t}^{2}+{{{\epsilon }}}_{t}$$

where *Y*_*t*_ is one of three outcome variables (the logarithm of current smokers below the age of 25, the logarithm of current smoking prevalence for such a group and the quit ratio in the 45–59 age group, respectively), *T*_*t*_ is a time trend, *I*_*t*_ is a time trend since FCTC ratification, *X*_*t*_ is a dichotomous variable that identifies the FCTC ratification period and *ϵ*_*t*_ is the error. The difference between the models is how they adjust for nonlinearity in the before and after ratification periods. The parameters *β*_0_–*β*_3_ adjust the before ratification trends, while *β*_4_–*β*_6_ adjust the after intervention trends. Concretely, *β*_0_ corresponds to the (log) number of smokers/prevalence or quit ratio smokers at the beginning of the period considered (for example, 10 years before ratification). *β*_1_, *β*_2_ and *β*_3_, as appropriate, explain the slope of the outcome variable in the before treatment period. *β*_4_ represents an immediate level change at the ratification year. Finally, as appropriate, *β*_5_ and *β*_6_ represent the change in slope between the before and after ratification period (Supplementary Fig. 1). Therefore, to analyze the effect of the FCTC, we must find statistical significance in *β*_4_ that supports an immediate effect on ratification or statistical significance in *β*_5_ or *β*_6_ for the existence of an effect over time^46^. Because dependent variables are in logarithm, parameters show relative percentage changes.

We estimated the models using ordinary least squares with the Newey–West correction of standard errors to account for autocorrelation and heteroskedasticity^13^. We used Stata 17 to run these models. Stata’s margins command was used to calculate the average before ratification trend, average change in trend and cumulative effect of the agreement 10 years after ratification. The models with the best before ratification fit were selected using the Akaike information criterion (AIC) (Supplementary Tables 6–8)^46^. The AIC is a well-known method to establish the prediction error of models and, therefore, to compare their relative quality^49^. To calculate the average before ratification trend, the best-fit model was derived for the variable ${T}_{t}$ in the before ratification period. Then, to calculate the average change in trend, the best-fit model was derived for the *I*_*t*_ variable in the after ratification period. Finally, to calculate the FCTC effect 10 years after ratification, the best-fit model was derived for the ${X}_{t}$ variable, conditional on the ${I}_{t}$ variable taking a value of 10.

The outcome of the number of smokers is an aggregation of current smokers across each country within groups (for example, the high-income group). Similarly, we aggregated current and former smokers according to groups of countries and population age groups according to the year of each country’s ratification. Finally, we estimated the logarithms of each outcome to quantify relative changes over time. Given that we made multiple comparisons, we considered statistical significance differences exceeding 95% probability (*P* < 0.05).

We made five notable enhancements to previous ITSAs that assessed the relationship between smoking outcomes and the adoption of the FCTC^8^. First, we focused on the age-appropriate number of smokers, a more directly relevant outcome than per capita cigarette consumption. Second, we considered ITSA models with linear and nonlinear trends and selected the ones that better matched the before and after ratification trends. Third, given that the ITSA requires the intervention to be applied at the same time to the treated units^50^ (that is, countries that ratified the FCTC), we realigned the data, centering each country’s number of current smokers (and therefore, prevalence) on the year of each country’s FCTC ratification (*T*_0_). Hence, the number of current smokers was aggregated according to country income group (for example, low-income), considering each country’s ratification year and constructing the time series backward and forward, accordingly. By doing this, we aggregated countries with different ratification years and considered each country’s time to ratification and time after ratification. The alternative would be to consider an artificially fixed year of ratification and assess the effects from there, even when countries ratified later. Previous analyses considered 2003, when the FCTC was adopted, as the primary intervention date. However, only Fiji, Malta, Norway, the Seychelles and Sri Lanka ratified it in 2003, and the FCTC entered force in 2005 (refs. ^8,51^).

Fourth, the number of before- and after-FCTC ratification periods considered can affect the estimation of the before-FCTC trend and, consequently, the assessment of changes in before- and after-FCTC ratification periods^14^. For countries that ratified the FCTC in, for instance, 2006, it makes little sense to consider the trend in the number of current smokers since 1970 (ref. ^8^). We considered a before-FCTC ratification period of 10 years to capture the preexisting levels and trends of a sufficiently long but near period to ratification. In addition, we considered an after-FCTC ratification period of 10 years, which involved working with countries that ratified until 2010 (as the last year in the GBD smoking dataset is 2020). Thus, we excluded ten countries that ratified after 2010 (Andorra, Czechia, El Salvador, Ethiopia, Mozambique, Saint Kitts and Nevis, Tajikistan, Turkmenistan, Uzbekistan and Zimbabwe), representing 1.3% of the total global smokers and 2.5% of the total global population.

Fifth, given that we worked with the number of current smokers (and associated prevalence) for groups of countries according to income level, the countries included in each income group must be kept constant. In sensitivity analyses, we considered the maximum possible before intervention period (that is, 13 years because the dataset starts in 1990 and the first countries ratified the FCTC in 2003), which yielded similar results (data not shown). Sensitivity analyses also included examining only 65 countries with higher-quality surveys (at least 10 years of surveys), which yielded similar results (data shown in Supplementary Table 2). Finally, we considered 2005 (when the FCTC became legally binding) as the starting year for countries that ratified before 2005; this also yielded similar results (data not shown).

### Ethics and inclusion statement

The study used de-identified and compiled data with no individual identifiers; thus, no institutional review board review was needed.

Data for LICs, LMICs, higher- and middle-income countries and HICs were used for this study. Two of the authors (G.P. and M.F.M.) are based in Chile, a developing HIC; the others are based in a developed HIC. We fully endorse the Nature Portfolio journals’ guidance on authorship and inclusion. This research is globally relevant, but especially relevant to LICs and LMICs because they bear a very high disease burden attributed to tobacco, which puts enormous pressure on already strained health systems and relatively poorer individuals in those countries.

### Reporting summary

Further information on research design is available in the Nature Portfolio Reporting Summary linked to this article.

## Online content

Any methods, additional references, Nature Portfolio reporting summaries, source data, extended data, supplementary information, acknowledgements, peer review information; details of author contributions and competing interests; and statements of data and code availability are available at 10.1038/s41591-024-02806-0.

### Supplementary information

Supplementary Information Supplementary Tables 1–9 and Fig. 1.

Reporting Summary

## Data Availability

The input data from the GBD are available at https://ghdx.healthdata.org/record/ihme-data/gbd-2019-smoking-tobacco-use-prevalence-1990–2019.

## Code availability

All code used in this study is available from https://osf.io/tqdg4/?view_only=8266983fff444cf2abb5b89344e460ac.
