# Supplementary material for: Reductions in smoking due to ratification of the Framework Convention for Tobacco Control in 171 countries
Source: Nat Med. 2024 Feb 6;30(3):683–9. doi: 10.1038/s41591-024-02806-0 (PMC10957467; doi:10.1038/s41591-024-02806-0)
Supplement: Supplementary file 1 — Supplementary Tables 1–9 and Fig. 1. [file 41591_2024_2806_MOESM1_ESM.pdf]

nature medicine

Article https://doi.org/10.1038/s41591-024-02806-0

Reductions in smoking due to ratification of the Framework Convention for Tobacco Control in 171 countries

In the format provided by the authors and unedited

Supplementary Appendix

Country Name	Pre-Ratification Period		Year of WHO FCTC Ratification	Post-Ratification Period		Tax burden most sold brand in 2008 (% of retail price)	Tax burden change 2008/2012 in percentage points	Number of available surveys	-
	Number of current smokers (thousands)	Prevalence of current smoking		Number of current smokers (thousands)	Prevalence of current smoking				Data Quality (*)
**Low Income Countries**									
Afghanistan	1.163	7.7%	2010	2,119	9.3%	8%	-5	3	Poor
Burkina Faso	820	9.0%	2006	1,032	8.9%	31%	2	7	Medium
Burundi	398	9.5%	2005	506	8.5%	59%	-5	3	Poor
Central African Republic	200	8.3%	2005	230	7.4%	-	-	0	Poor
Chad	600	11.3%	2006	710	9.3%	25%	0	5	Medium
Democratic People's Republic of Korea	4.580	24.5%	2005	5,028	23.3%	-	-	1	Poor
Democratic Republic of the Congo	3.020	9.1%	2005	4,137	9.2%	29%	11	2	Poor
Gambia	115	11.9%	2007	127	9.7%	52%	-14	-	-
Guinea	548	9.9%	2007	629	8.9%	10%	1	2	Poor
Guinea-Bissau	27	3.0%	2008	50	4.3%	6%	0	-	-
Liberia	156	7.2%	2009	199	6.4%	12%	8	6	Medium
Madagascar	1.852	18.1%	2004	2,125	14.8%	67%	9	5	Medium
Mali	723	10.2%	2005	1,137	11.0%	17%	11	5	Medium
Niger	711	10.2%	2005	858	8.5%	28%	3	9	Medium
Rwanda	508	9.5%	2005	743	10.2%	33%	1	7	Medium
Sierra Leone	688	19.7%	2009	849	16.8%	13%	0	6	Medium
Sudan	2.166	12.0%	2005	2,482	10.5%	68%	0	6	Medium
Syrian Arab Republic	2.779	24.4%	2004	3,387	23.5%	33%	0	6	Medium
Togo	305	9.4%	2005	346	7.8%	20%	2	5	Medium
Uganda	2.055	12.7%	2007	2,293	10.2%	59%	-24	>10	High
Yemen	2.012	15.5%	2007	3,001	16.5%	55%	-2	7	Medium
**Middle-Low Income Countries**									
Algeria	3.511	14.4%	2006	4,529	15.2%	52%	-2	3	Poor
Angola	882	8.5%	2007	1,383	9.0%	23%	0	1	Poor
Bangladesh	18.714	20.8%	2004	23,548	21.4%	47%	7	>10	High
Belize	21	12.1%	2005	31	12.5%	57%	-20	-	-
Benin	351	8.2%	2005	377	6.1%	7%	1	8	Medium
Bhutan	55	11.6%	2004	63	11.1%	-	-	-	-
Bolivia	559	9.3%	2005	903	11.6%	41%	-2	7	Medium
Cabo Verde	21	6.4%	2005	22	5.6%	10%	2	-	-
Cambodia	1.788	20.0%	2005	2,095	18.6%	17%	0	>10	High
Cameroon	968	9.3%	2006	1,216	7.8%	32%	10	6	Medium
Comoros	53	13.3%	2006	60	12.4%	42%	9	-	-

Congo	195	8.2%	2007	353	11.1%	32%	0	4	Poor	
Côte d'Ivoire	1.785	13.5%	2010	1,912	11.3%	25%	0	3	Poor	
Djibouti	82	18.2%	2005	142	21.1%	24%	5	-	-	
Egypt	9.132	17.9%	2005	12,900	20.0%	59%	13	>10	High	
Eswatini	66	9.2%	2006	61	7.7%	-	-	-	-	
Ghana	633	4.9%	2004	982	5.5%	27%	-4	12	High	
Honduras	664	15.4%	2005	825	13.8%	45%	-11	7	Medium	
India	122.598	16.2%	2004	136,614	14.3%	46%	-4	>10	High	
Islamic Republic of Iran	6.043	11.6%	2005	8,997	14.1%	3%	0	6	Medium	
Kenya	2.919	14.3%	2004	2,980	10.7%	76%	-28	>10	High	
Kiribati	28	44.8%	2005	36	46.0%	50%	2	-	-	
Kyrgyzstan	735	18.8%	2006	965	21.3%	38%	-6	>10	High	
Lao People's Democratic Republic	725	18.9%	2006	1,040	21.2%	12%	8	7	Medium	
Lesotho	161	12.8%	2005	231	16.9%	29%	17	-	-	
Mauritania	397	22.5%	2005	469	20.4%	9%	-5	6	Medium	
Federated States of Micronesia	35	44.4%	2005	38	47.5%	67%	-7	-	-	
Mongolia	420	22.9%	2004	591	26.1%	40%	10	9	Medium	
Myanmar	9.419	26.8%	2004	9,195	23.1%	25%	25	11	High	
Nepal	5.026	29.0%	2006	4,445	20.7%	24%	6	>10	High	
Nicaragua	586	15.4%	2008	721	15.4%	20%	9	5	Medium	
Nigeria	4.661	5.6%	2005	4,946	4.4%	21%	0	10	Medium	
Pakistan	16.238	17.3%	2004	18,091	14.4%	52%	8	10	Medium	
Papua New Guinea	1.602	39.8%	2006	2,079	36.3%	42%	-5	5	Medium	
Philippines	18.098	31.1%	2005	19,545	26.5%	29%	0	>10	High	
Samoa	38	29.2%	2005	38	27.0%	63%	-3	-	-	
Sao Tome and Principe	5	4.6%	2006	6	4.5%	25%	0	-	-	
Senegal	969	14.5%	2005	891	10.2%	27%	3	10	Medium	
Solomon Islands	89	30.0%	2004	122	31.8%	-	-	-	-	
Sri Lanka	2.564	17.2%	2003	2,332	14.0%	72%	3	>10	High	
Timor-Leste	205	33.5%	2004	258	33.7%	-	-	-	-	
Tunisia	2.029	23.9%	2010	2,159	22.9%	85%	-7	8	Medium	
Ukraine	14.185	31.9%	2006	12,062	29.1%	49%	18	>10	High	
United Republic of Tanzania	2.943	12.3%	2007	2,867	9.0%	35%	-7	9	Medium	
Vanuatu	36	26.7%	2005	43	24.2%	56%	-3	-	-	
Viet Nam	15.372	25.1%	2004	17,039	23.1%	32%	0	>10	High	
Zambia	1.042	14.8%	2008	1,588	15.4%	47%	-10	8	Medium	
**Upper Middle-Income Countries**										
Albania	570	21.8%	2006	681	27.2%	50%	11	-	-	
Armenia	731	26.2%	2004	727	26.6%	22%	3	-	-	
Azerbaijan	1.357	20.6%	2005	1,737	22.2%	21%	-2	>10	High	
Belarus	2.769	30.5%	2005	2,544	29.3%	29%	7	>10	High	
Bosnia and Herzegovina	1.231	33.9%	2009	1,159	35.8%	57%	18	>10	High	
Botswana	227	18.4%	2005	297	19.1%	43%	7	-	-	
Brazil	31.625	22.7%	2005	23,728	14.3%	57%	6	>10	High	
Bulgaria	2.790	38.5%	2005	2,407	35.6%	85%	-2	>10	High	

China	290.765	26.9%	2005	316,848	26.6%	41%	9	>10	High
Colombia	6.260	19.4%	2008	4,941	13.1%	34%	16	8	Medium
Costa Rica	560	17.2%	2008	456	12.0%	42%	17	>10	High
Dominica	6	10.6%	2006	6	10.0%	26%	-2	-	-
Ecuador	1.235	13.0%	2006	1,809	15.0%	64%	9	8	Medium
Equatorial Guinea	50	11.5%	2005	92	12.7%	21%	7	-	-
Fiji	185	29.9%	2003	188	27.2%	50%	-9	-	-
Gabon	106	11.1%	2009	142	11.8%	17%	3	-	-
Georgia	998	24.6%	2006	915	26.5%	49%	-2	>10	High
Grenada	10	12.1%	2007	10	11.5%	-	-	-	-
Guatemala	981	13.3%	2005	1,334	13.2%	52%	-3	9	Medium
Guyana	82	14.1%	2005	85	14.2%	28%	-3	-	-
Iraq	3.680	18.6%	2008	5,006	17.9%	1%	0	5	Medium
Jamaica	365	17.7%	2005	345	15.0%	44%	2	-	-
Jordan	860	25.7%	2004	1,552	29.2%	77%	-1	>10	High
Kazakhstan	3.162	25.1%	2007	3,355	24.5%	20%	10	>10	High
Lebanon	825	29.3%	2005	1,178	31.9%	44%	-1	8	Medium
Libya	600	15.6%	2005	930	18.6%	24%	-8	6	Medium
Malaysia	4.775	26.4%	2005	5,540	24.2%	54%	3	>10	High
Maldives	41	21.0%	2004	64	22.8%	30%	19	-	-
Marshall Islands	8	21.6%	2004	9	23.1%	54%	-14	-	-
Mauritius	225	22.9%	2004	232	21.4%	81%	-8	-	-
Mexico	17.522	23.2%	2004	16,345	18.1%	61%	7	>10	High
Montenegro	206	37.9%	2006	203	36.8%	45%	36	-	-
Namibia	252	19.2%	2005	279	17.7%	43%	5	-	-
Niue	<0.1	20.5%	2005	<0.1	19.5%	76%	-9	-	-
North Macedonia	638	35.9%	2006	694	36.9%	72%	-1	-	-
Panama	291	13.2%	2004	269	9.7%	37%	20	7	Medium
Paraguay	828	21.4%	2006	774	15.8%	16%	2	8	Medium
Peru	2.640	13.8%	2004	3,186	13.8%	41%	1	>10	High
Republic of Moldova	828	22.9%	2009	777	22.9%	22%	22	>10	High
Romania	6.062	30.6%	2006	5,135	28.0%	72%	1	>10	High
Russian Federation	45.057	33.7%	2008	39,843	30.9%	30%	10	>10	High
Saint Lucia	17	14.4%	2005	18	12.8%	30%	0	-	-
Saint Vincent and the Grenadines	12	13.1%	2010	12	12.2%	15%	3	-	-
Serbia	2.828	33.9%	2006	2,756	34.0%	62%	19	>10	High
South Africa	8.533	24.3%	2005	9,527	23.4%	48%	2	>10	High
Suriname	96	25.0%	2008	97	21.3%	57%	3	-	-
Thailand	12.400	23.8%	2004	13,100	22.1%	64%	6	>10	High
Tonga	22	29.7%	2005	21	27.0%	43%	9	-	-
Turkey	17.026	32.6%	2004	18,357	30.1%	73%	7	>10	High
Tuvalu	2	29.9%	2005	3	31.0%	-	-	-	-
Venezuela (Bolivarian Republic of)	3.234	17.7%	2006	3,564	15.6%	71%	0	5	Medium
**High Income Countries**									
Antigua and Barbuda	5	7.6%	2006	6	8.1%	15%	0	-	-

Australia	3.633	22.8%	2004	3,219	17.4%	62%	-2	>10	High
Austria	2.394	33.6%	2005	2,262	29.8%	74%	0	>10	High
Bahamas	20	7.4%	2009	24	7.6%	31%	-5	-	-
Bahrain	82	13.7%	2007	179	16.0%	33%	-13	-	-
Barbados	24	10.9%	2005	22	9.1%	48%	0	-	-
Belgium	2.596	28.6%	2005	2,300	23.9%	77%	-1	>10	High
Brunei Darussalam	47	18.9%	2004	52	16.2%	54%	14	-	-
Canada	6.870	26.4%	2004	5,541	18.8%	65%	0	>10	High
Chile	4.771	38.6%	2005	5,106	35.7%	76%	5	5	Medium
Cook Islands	6	38.6%	2004	5	33.0%	46%	25	-	-
Croatia	1.281	31.8%	2008	1,255	32.0%	61%	10	>10	High
Cyprus	258	32.8%	2005	331	33.3%	72%	4	-	-
Denmark	1.650	35.6%	2004	1,187	24.4%	73%	6	>10	High
Estonia	364	29.3%	2005	345	28.9%	77%	0	-	-
Finland	1.114	24.4%	2005	982	20.5%	77%	2	>10	High
France	16.431	31.6%	2004	16,488	29.7%	80%	-1	>10	High
Germany	21.397	29.0%	2004	19,187	25.6%	76%	-3	>10	High
Greece	3.854	38.6%	2006	3,681	37.3%	73%	9	>10	High
Hungary	3.089	33.9%	2004	2,898	32.1%	74%	10	>10	High
Iceland	56	24.3%	2004	46	17.2%	58%	-1	-	-
Ireland	977	29.5%	2005	961	24.6%	80%	-1	-	-
Israel	1.240	24.5%	2005	1,284	20.3%	72%	5	>10	High
Italy	12.963	24.9%	2008	12,258	22.3%	75%	0	>10	High
Japan	35.746	30.8%	2004	28,010	23.6%	63%	1	>10	High
Kuwait	415	26.1%	2006	659	24.7%	36%	-7	9	Medium
Latvia	652	30.4%	2005	604	31.5%	72%	8	-	-
Lithuania	812	26.2%	2004	761	26.9%	68%	8	-	-
Luxembourg	110	29.2%	2005	108	24.0%	70%	0	-	-
Malta	91	26.7%	2003	81	22.3%	76%	1	-	-
Nauru	4	45.6%	2004	3	44.1%	-	-	-	-
Netherlands	4.330	31.2%	2005	3,573	24.3%	74%	-1	>10	High
New Zealand	833	25.8%	2004	736	20.3%	70%	4	>10	High
Norway	1.336	34.9%	2003	997	23.9%	73%	0	>10	High
Oman	160	9.3%	2005	259	10.1%	33%	-11	7	Medium
Palau	4	23.8%	2004	4	23.0%	57%	0	-	-
Poland	10.481	30.9%	2006	9,750	28.2%	94%	-14	>10	High
Portugal	2.361	25.1%	2005	2,356	24.2%	80%	-4	>10	High
Qatar	87	18.5%	2004	286	21.0%	33%	-11	-	-
Republic of Korea	11.546	28.8%	2005	10,756	24.1%	62%	0	9	Medium
San Marino	6	26.0%	2004	5	20.1%	-	-	-	-
Saudi Arabia	2.070	13.3%	2005	3,470	15.1%	33%	-11	4	Poor
Seychelles	16	25.2%	2003	18	23.8%	76%	-9	-	-
Singapore	578	17.2%	2004	714	16.2%	67%	-2	10	Medium
Slovakia	1.377	29.3%	2004	1,278	26.3%	85%	-2	>10	High
Slovenia	412	23.0%	2005	465	25.2%	75%	4	-	-
Spain	12.561	34.0%	2005	13,735	33.0%	77%	1	>10	High

Sweden	12.561	18.8%	2005	1,218	14.5%	72%	2	>10	High
Trinidad and Tobago	190	17.8%	2004	214	18.5%	37%	-4	-	-
United Arab Emirates	557	20.8%	2005	1,442	21.2%	31%	-6	7	Medium
United Kingdom	13.898	27.1%	2004	12,367	22.4%	.77	4	>10	High
Uruguay	874	32.0%	2004	748	26.1%	.66	3	>10	High

Table A1. Average number of current smokers and prevalence of current smoking in the pre-FCTC and post-FCTC periods (total population); year of WHO FCTC ratification; total tax burden on most sold brand (as percentage of retail price) and tobacco tax change in the 2008-2012 period; number of available surveys.

(*) High-quality data refers to countries with more than 10 nationally representative surveys; medium-quality data refers to countries with 5-10 (both included) nationally representative surveys; poor-quality refers to countries with less than 5 nationally representative surveys.

Region (number of countries)	Number smokers below age 25 years; and current/former smokers ages 45-59 in ratification year (millions)	Average change in trends after ratification (95%CI) Number of best fitted model*		
		Logarithm of the number of smokers (%) below age 25 years	Logarithm of Prevalence (%) below age 25 years	Logarithm of Quit Ratio (%) age 45-59
**World (57)**	118; 127/66	-0.9% (-1.1, -0.6)[2]	-0.6% (-0.8, -0.4)[2]	0.1% (0.1, 0.2)[1]
**By Income Group**				
**High (24)**	28; 40/30	-0.2% (-0.4, -0.1)[4]	0.1% (-0.4, 0.5)[2]	0.2% (0.1, 0.2)[1]
**Upper-middle (19)**	39; 36/22	-6.0% (-11.0, -0.1)[5]	-1.5% (-2.1, -0.9)[4]	0.6% (0.4, 0.7)[4]
**Lower-middle (13)**	50; 51/14	-0.9% (-1.1, -0.8)[4]	-0.8% (-0.9, -0.7)[4]	0.0% (-0.1, 0.2)[1]
**Low (1)**	1; 0.3/0.2	-7.6% (-10.6, -4.7)[5]	-8.5% (-11.5, -5.4)[5]	0.2% (0.0, 0.3)[4]
**By Tax Change**				
**High-tax change (11)**	20; 23/8	-3.5% (-4.5, -2.4)[4]	-2.5 (-3.3, -1.7)[4]	0.4% (0.3, 0.5)[4]
**Low-tax change (46)**	98; 104/58	-1.2% (-1.4, -1.0)[3]	-1.0 (-1.2, -0.9)[3]	-0.2% (-0.3, -0.1)[1]

Table A2. Average change in trends after WHO FCTC ratification in logarithm of number of smokers and prevalence for population below age 25 years and the logarithm of the quit ratio for the population between 45 and 59 years old by country groupings, considering only "High-quality" datasets.
FCTC=WHO Framework Convention for Tobacco Control. A list of the five possible ITSA models is provided in the methods.
Model 1 corresponds to pre- and post-intervention linear trends; model 2 corresponds to a pre-intervention polynomic quadratic trend and a post-intervention linear trend; model 3 corresponds to pre- and post-intervention polynomic quadratic trends; model 4 corresponds to a pre-intervention polynomic cubic trend and a post-intervention linear trend; model 5 corresponds to a pre-intervention polynomic cubic trend and a post-intervention polynomic quadratic trend.

Region (number of countries)*Number of best fitted model**	Change in logarithm of number of smokers on population below age 25 years (95%CI)		
	Average pre-ratification trend (%)	Change in level at ratification (%)	Average change in trend after ratification (%)
**World (170)**[5]	0.5% (0.4, 0.5)	-0.5% (-1.4, 0.3)	-1.6% (-3.1, -0.1)
By Income Group			
**High (52)**[4]	-1.7% (-1.7, -1.6)	-0.7% (-1.2, -0.3)	-0.1% (-0.3, 0.1)
**Upper-middle (50)**[5]	-0.0% (-0.2, 0.2)	-1.0% (-3.0, 1.0)	-3.3% (-7.2, 0.5)
**Lower-middle (47)**[4]	1.9% (1.9, 1.9)	-0.2% (-0.5, 0.2)	-0.8% (-1.0, -0.7)
**Low (21)**[5]	2.9% (2.8, 3.0)	-0.3% (-1.1, 0.4)	-6.0% (-8.1, -4.0)
By Tax Change Group			
**High-tax change (23)**[4]	0.4% (0.3, 0.6)	-1.1 (-3.1, 0.9)	-2.7% (-3.6, -1.8)
**Low-tax change (137)**[3]	0.5% (0.5, 0.5)	-0.3 (-0.6, 0.0)	-1.2% (-1.4, -1.0)

Table A3. Evolution of logarithm of number of smokers by country and tax change groupings.
Underlined excludes China. FCTC=WHO Framework Convention for Tobacco Control. A list of the five possible ITSA models is provided in the methods.
Model 1 corresponds to pre- and post-intervention linear trends; model 2 corresponds to a pre-intervention polynomic quadratic trend and a post-intervention linear trend; model 3 corresponds to pre- and post-intervention polynomic quadratic trends; model 4 corresponds to a pre-intervention polynomic cubic trend and a post-intervention linear trend; model 5 corresponds to a pre-intervention polynomic cubic trend and a post-intervention polynomic quadratic trend.

Region (number of countries) $^{\text{Number of best fitted model*}}$	Change in logarithm of prevalence on population below age 25 years (95%CI)		
	Average pre-ratification trend (%)	Change in level at ratification (%)	Average change in trend after ratification (%)
World (170)^{3}	-1.0% (-1.0, -0.9)	-0.3% (-0.7, 0.1)	-0.8% (-1.0, -0.5)
**By Income Group**			
High (52)^{5}	-1.1% (-1.1, -1.0)	-0.1% (-0.4, 0.3)	1.3% (0.6, 1.9)
Upper-middle (50)^{4}	-0.5% (-0.6, -0.4)	-0.5% (-1.5, 0.5)	-1.0% (-1.5, -0.6)
Lower-middle (47)^{4}	-0.1% (-0.1, -0.1)	-0.1% (-0.4, 0.2)	-0.7% (-0.8, -0.5)
Low (21)^{5}	-0.3% (-0.4, -0.2)	-0.4% (-1.0, 0.3)	-4.0% (-5.6, -2.4)
**By Tax Change Group**			
High-tax change (23)^{4}	-0.2% (-0.4, -0.1)	-1.0% (-2.6, 0.6)	-2.1% (-2.8, -1.4)
Low-tax change (137)^{5}	-1.1% (-1.1, -1.0)	-0.2% (-0.5, 0.1)	-0.7% (-1.3, -0.2)

Table A4. Evolution of logarithm of prevalence for population below age 25 years by country and tax change groupings.

Underlined excludes China. FCTC=WHO Framework Convention for Tobacco Control. A list of the five possible ITSA models is provided in the methods.

Model 1 corresponds to pre- and post-intervention linear trends; model 2 corresponds to a pre-intervention polynomic quadratic trend and a post-intervention linear trend; model 3 corresponds to pre- and post-intervention polynomic quadratic trends; model 4 corresponds to a pre-intervention polynomic cubic trend and a post-intervention linear trend; model 5 corresponds to a pre-intervention polynomic cubic trend and a post-intervention polynomic quadratic trend.

Region (number of countries) *Number of best fitted model**	**Change in logarithm of quit ratio** on population between 45 and 59 years old (95%CI)		
	Average pre-ratification trend (%)	Change in level at ratification (%)	Average change in trend after ratification (%)
**World (170)** [1]	0.8% (0.7, 0.8)	0.3% (0.1, 0.6)	0.1% (0.1, 0.2)
**By Income Group**			
**High** (52)[1]	0.5% (0.4, 0.5)	0.0% (-0.3, 0.3)	0.2% (0.1, 0.2)
**Upper-middle (50)**[5]	0.2% (0.0, 0.3)	0.3% (-0.2, 0.9)	-0.2% (-1.0, 0.6)
**Lower-middle** (47)[1]	1.3% (1.3, 1.4)	-0.7% (-1.3, -0.1)	0.1% (0.0, 0.3)
**Low** (21)[1]	0.9% (0.8, 0.9)	0.2% (-0.1, 0.5)	-0.2% (-0.2, -0.1)
**By Tax Change Group**			
**High-tax change** (23)[4]	-1.7% (-1.8, -1.5)	0.4% (0.1, 0.6)	0.5% (0.4, 0.6)
**Low-tax change** (137)[1]	0.9% (0.9, 0.9)	-0.3% (-0.5, -0.0)	-0.1% (-0.2, -0.1)

Table A5. Evolution of logarithm of the quit ratio for the population between 45 and 59 years old by country and tax change groupings.

Underlined excludes China. FCTC=WHO Framework Convention for Tobacco Control. A list of the five possible ITSA models is provided in the methods.

Model 1 corresponds to pre- and post-intervention linear trends; model 2 corresponds to a pre-intervention polynomic quadratic trend and a post-intervention linear trend; model 3 corresponds to pre- and post-intervention polynomic quadratic trends; model 4 corresponds to a pre-intervention polynomic cubic trend and a post-intervention linear trend; model 5 corresponds to a pre-intervention polynomic cubic trend and a post-intervention polynomic quadratic trend.

**Region (number of countries)**	**<u>Akaike Information Criterion for logarithm of smokers (Bold for mínimum AIC)</u>**			
	<u>Model</u>			
	2	*3*	*4*	*5*
**<u>World (170)</u>**	-201.19	-200.62	-200.01	**-201.51**
**By Income Group**				
**High (52)**	-190.14	-200.13	**-200.47**	-198.48
**<u>Upper-middle (50)</u>**	-119.11	-152.09	-160.03	**-160.36**
**Lower-middle (47)**	-141.98	-208.53	**-216.01**	-215.89
**Low (21)**	-144.48	-164.66	-157.5	**-187.14**
**By Tax Change Group**				
**High-tax change (23)**	-101.18	-139.36	**-146.24**	-144.81
**Low-tax change (137)**	-167.36	**-221.04**	-210.46	-220.33

Table A6. Results for the Akaike Information Criterion of non-linear models (2)-(5) for the logarithm of the number of current smokers prevalence for population below age 25 years, by income and tax change groupings.

<u>Underlined</u> excludes China.

Model 1 corresponds to pre- and post-intervention linear trends; model 2 corresponds to a pre-intervention polynomic quadratic trend and a post-intervention linear trend; model 3 corresponds to pre- and post-intervention polynomic quadratic trends; model 4 corresponds to a pre-intervention polynomic cubic trend and a post-intervention linear trend; model 5 corresponds to a pre-intervention polynomic cubic trend and a post-intervention polynomic quadratic trend.

**Region (number of countries)**	**Akaike Information Criterion for logarithm of prevalence (Bold for minimum AIC)** Model			
	2	*3*	*4*	*5*
**World (170)**	-205.23	**-219.65**	-219.31	-217.70
**By Income Group**				
**High (52)**	-182.28	-234.50	-222.08	**-236.38**
**Upper-middle (50)**	-134.66	-169.66	**-174.53**	-172.75
**Lower-middle (47)**	-136.22	-205.02	**-222.37**	-221.24
**Low (21)**	-148.45	-185.68	-174.69	**-198.70**
**By Tax Change Group**				
**High-tax change (23)**	-123.87	-152.42	**-155.55**	-153.67
**Low-tax change (137)**	-169.23	-243.16	-240.89	**-245.86**

Table A7. Results for the Akaike Information Criterion of non-linear models (2)-(5) for the logarithm of prevalence of current smoking prevalence for population below age 25 years, by income and tax change groupings.
Underlined excludes China.
Model 1 corresponds to pre- and post-intervention linear trends; model 2 corresponds to a pre-intervention polynomic quadratic trend and a post-intervention linear trend; model 3 corresponds to pre- and post-intervention polynomic quadratic trends; model 4 corresponds to a pre-intervention polynomic cubic trend and a post-intervention linear trend; model 5 corresponds to a pre-intervention polynomic cubic trend and a post-intervention polynomic quadratic trend.

Region (number of countries)	Akaike Information Criterion for logarithm of the quit ratio (Bold for mínimum AIC)			
	Model			
	2	*3*	*4*	*5*
World (170)	Linear pre-ratification trend			
**By Income Group**				
High (52)	Linear pre-ratification trend			
Upper-middle (50)	-140.45	-205.62	-207.64	**-208.70**
Lower-middle (47)	Linear pre-ratification trend			
Low (21)	Linear pre-ratification trend			
**By Tax Change Group**				
High-tax change (23)	-123.70	-190.89	**-213.84**	-211.96
Low-tax change (137)	Linear pre-ratification trend			

Table A8. Results for the Akaike Information Criterion of non-linear models (2)-(5) for the logarithm of the quit ratio for the population between 45 and 59 years old, by income and tax change groupings.
Underlined excludes China.
Model 1 corresponds to pre- and post-intervention linear trends; model 2 corresponds to a pre-intervention polynomic quadratic trend and a post-intervention linear trend; model 3 corresponds to pre- and post-intervention polynomic quadratic trends; model 4 corresponds to a pre-intervention polynomic cubic trend and a post-intervention linear trend; model 5 corresponds to a pre-intervention polynomic cubic trend and a post-intervention polynomic quadratic trend.

Region<br><br>[Best fitted model]	Number of countries<br><br>(M of smokers at ratification year)	Former smokers achieved<br><br>on population between 45 and 59 years old (95%CI)		
		Number of former smokers (thousands)	Relative (%) decrease in former smokers	Impact if all group had high tax change (thousands)
World [1]	170 (151.5)	2,000<br>(1,394; 2,611)	2.1%<br>(1.4; 2.7)	5,238<br>(3,983; 6,866)
**By Income Group**				
High [1]	52 (45.3)	607<br>(420; 796)	1.7%<br>(1.1; 2.2)	1,964<br>(1,527; 2,569)
Upper-middle [5]	50 (40.4)	258<br>(-2,141; 2,983)	0.9%<br>(-7.1; 9.9)	1,577<br>(1,206; 2,139)
Lower-middle [1]	47 (61)	348<br>(44; 653)	1.3%<br>(0.2; 2.4)	1,472<br>(880; 1,932)
Low [1]	50 (40.4)	-44<br>(-68; -20)	-1.4%<br>(-2.1; -0.6)	173<br>(130; 237)
**By Tax Change**				
High-tax change [4]	23 (26)	599<br>(433; 767)	5.5%<br>(4.0; 7.1)	599<br>(433; 767)
Low-tax change [1]	137 (124)	-820<br>(-1,175; -463)	-1.0%<br>(-1.4; -0.5)	4,510<br>(3,357; 6,575)

Table A9. Evolution of the number of former smokers in population 45-59 years old after 10 years of WHO FCTC ratification. China is excluded from income or tax change categories. FCTC= WHO Framework Convention for Tobacco Control.

Underlined excludes China.

Model 1 corresponds to pre- and post-intervention linear trends; model 2 corresponds to a pre-intervention polynomic quadratic trend and a post-intervention linear trend; model 3 corresponds to pre- and post-intervention polynomic quadratic trends; model 4 corresponds to a pre-intervention polynomic cubic trend and a post-intervention linear trend; model 5 corresponds to a pre-intervention polynomic cubic trend and a post-intervention polynomic quadratic trend.

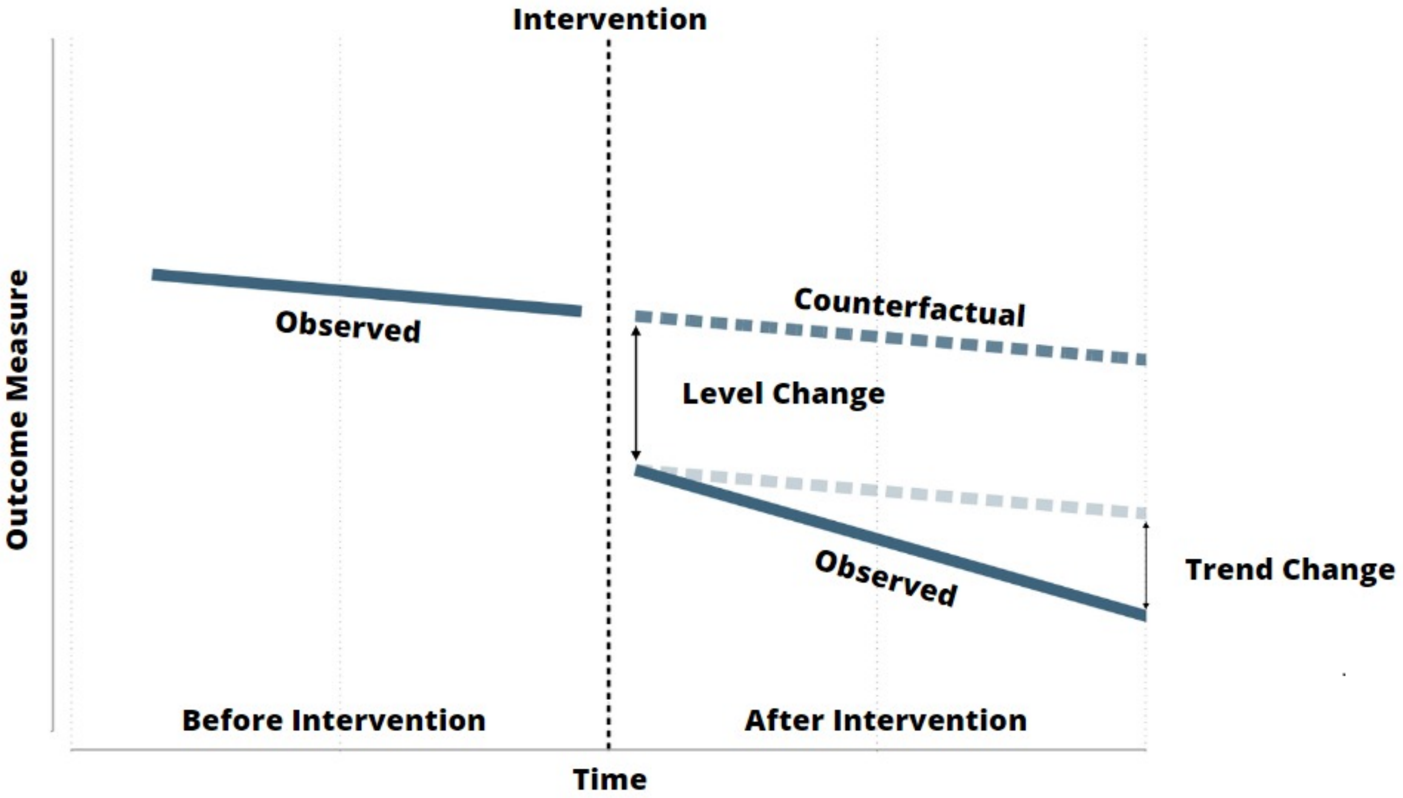

Figure A1. Graphic interpretation of the Interrupted Time Series methodology with a linear model.
