# Supplementary material for: Reductions in smoking due to ratification of the Framework Convention for Tobacco Control in 171 countries
Source: Nat Med. 2024 Feb 6;30(3):683–9. doi: 10.1038/s41591-024-02806-0 (PMC10957467; doi:10.1038/s41591-024-02806-0)
Supplement: Supplementary file 2 — Reporting Summary [file 41591_2024_2806_MOESM2_ESM.pdf]

nature portfolio

Corresponding author(s): Guillermo Paraje

Last updated by author(s): Nov 9, 2023

Reporting Summary

Nature Portfolio wishes to improve the reproducibility of the work that we publish. This form provides structure for consistency and transparency in reporting. For further information on Nature Portfolio policies, see our Editorial Policies and the Editorial Policy Checklist.

Statistics

For all statistical analyses, confirm that the following items are present in the figure legend, table legend, main text, or Methods section.

n/a	Confirmed	
☐	☒	The exact sample size (*n*) for each experimental group/condition, given as a discrete number and unit of measurement
☐	☒	A statement on whether measurements were taken from distinct samples or whether the same sample was measured repeatedly
☐	☒	The statistical test(s) used AND whether they are one- or two-sided<br>*Only common tests should be described solely by name; describe more complex techniques in the Methods section.*
☐	☒	A description of all covariates tested
☐	☒	A description of any assumptions or corrections, such as tests of normality and adjustment for multiple comparisons
☐	☒	A full description of the statistical parameters including central tendency (e.g. means) or other basic estimates (e.g. regression coefficient) AND variation (e.g. standard deviation) or associated estimates of uncertainty (e.g. confidence intervals)
☐	☒	For null hypothesis testing, the test statistic (e.g. *F*, *t*, *r*) with confidence intervals, effect sizes, degrees of freedom and *P* value noted<br>*Give P values as exact values whenever suitable.*
☒	☐	For Bayesian analysis, information on the choice of priors and Markov chain Monte Carlo settings
☒	☐	For hierarchical and complex designs, identification of the appropriate level for tests and full reporting of outcomes
☒	☐	Estimates of effect sizes (e.g. Cohen's *d*, Pearson's *r*), indicating how they were calculated

Our web collection on statistics for biologists contains articles on many of the points above.

Software and code

Policy information about availability of computer code

Data collection	The input data used in the study comes from the GBD and is freely available at: https://www.healthdata.org/data-tools-practices/data-sources. Dataset and codes used in the analyses are available at https://osf.io/tqdg4/?view_only=8266983fff444cf2abb5b89344e460ac
Data analysis	We have used Stata 17 MP for all the statistical analyses. No other software was used. All codes used in this study are available from https://osf.io/tqdg4/?view_only=8266983fff444cf2abb5b89344e460ac

For manuscripts utilizing custom algorithms or software that are central to the research but not yet described in published literature, software must be made available to editors and reviewers. We strongly encourage code deposition in a community repository (e.g. GitHub). See the Nature Portfolio guidelines for submitting code & software for further information.

Data

Policy information about availability of data

All manuscripts must include a data availability statement. This statement should provide the following information, where applicable:

- Accession codes, unique identifiers, or web links for publicly available datasets
- A description of any restrictions on data availability
- For clinical datasets or third party data, please ensure that the statement adheres to our policy

The input data from the GBD is available at: https://www.healthdata.org/data-tools-practices/data-sources. Dataset and codes used in the analyses are available at https://osf.io/tqdg4/?view_only=8266983fff444cf2abb5b89344e460ac

March 2021

Human research participants

Policy information about studies involving human research participants and Sex and Gender in Research.

Reporting on sex and gender	N/A
Population characteristics	N/A
Recruitment	N/A
Ethics oversight	N/A

Note that full information on the approval of the study protocol must also be provided in the manuscript.

Field-specific reporting

Please select the one below that is the best fit for your research. If you are not sure, read the appropriate sections before making your selection.

☐ Life sciences ☒ Behavioural & social sciences ☐ Ecological, evolutionary & environmental sciences

For a reference copy of the document with all sections, see nature.com/documents/nr-reporting-summary-flat.pdf

Behavioural & social sciences study design

All studies must disclose on these points even when the disclosure is negative.

Study description	The study uses cross-sectional data from national surveys from 171 countries, collected and compiled by GBD (Global Burden of Disease) at University of Washington. The study uses quantitative approaches (Interrupted Time-Series Analyses) to enquire about changes in levels and trends in current smoking population (age 10-24) and former smokers (age 45-59), after countries ratification of WHO Framework Convention on Tobacco Control (FCTC).
Research sample	We derived data on the number of smokers and smoking prevalence among individuals aged 10-24 years from datasets produced by the Global Burden of Disease, Injuries and Risk Factors (GBD) Study, which relied on 3,625 nationally representative surveys on tobacco use for 204 countries and territories from 1990-2020. Each survey has its own sampling strategy and population, which is not reported in the GBD study. We used this dataset as it is the most complete dataset with number of current and former smokers by age. The specific sample of countries used in the study is determined by the countries that ratified the FCTC.
Sampling strategy	Each survey of the 3,625 surveys originally considered by GBD has its own sampling strategy and population. We selected data for all countries that ratified the FCTC. Apart from this criterion, there is no sampling selection.<br>We also conducted separate analyses for countries where surveys were considered as "high-quality".
Data collection	The dataset provides annual estimates of smoking prevalence in 5-year age groups and the number of former smokers.<br>Characteristics of the surveys and imputation methods are described at lenght at "Reitsma MB, Kendrick PJ, Ababneh E, et al. Spatial, temporal, and demographic patterns in prevalence of smoking tobacco use and attributable disease burden in 204 countries and territories, 1990-2019: a systematic analysis from the Global Burden of Disease Study 2019. The Lancet 2021; 397(10292): 2337-60."<br>There is no primary data collection involved in the study.
Timing	There is no primary data collection in the study. Secondarty data was used. These annual data is from 1990 (initial year) to 2020 (final year).
Data exclusions	No data was excluded from the analyses.
Non-participation	Non-relevant for this study, as aggregate populaiton secondary data is used.
Randomization	Non-relevant for this study, as aggregate populaiton secondary data is used.

Reporting for specific materials, systems and methods

We require information from authors about some types of materials, experimental systems and methods used in many studies. Here, indicate whether each material, system or method listed is relevant to your study. If you are not sure if a list item applies to your research, read the appropriate section before selecting a response.

Materials & experimental systems

n/a	Involved in the study
☒	☐ Antibodies
☒	☐ Eukaryotic cell lines
☒	☐ Palaeontology and archaeology
☒	☐ Animals and other organisms
☒	☐ Clinical data
☒	☐ Dual use research of concern

Methods

n/a	Involved in the study
☒	☐ ChIP-seq
☒	☐ Flow cytometry
☒	☐ MRI-based neuroimaging
